# The role of the I_T_-state in D76N β_2_-microglobulin amyloid assembly: A crucial intermediate or an innocuous bystander?

**DOI:** 10.1074/jbc.RA120.014901

**Published:** 2020-07-13

**Authors:** Hugh I. Smith, Nicolas Guthertz, Emma E. Cawood, Roberto Maya-Martinez, Alexander L. Breeze, Sheena E. Radford

**Affiliations:** 1 Astbury Centre for Structural Molecular Biology, School of Molecular & Cellular Biology, Faculty of Biological Sciences, University of Leeds, Leeds, United Kingdom; 2 School of Chemistry, University of Leeds, Leeds, United Kingdom

**Keywords:** NMR, CD, β2-microglobulin, real-time folding, amyloid, D76N, folding intermediate, protein aggregation, protein folding, aggregation, biophysics, β2-microglobulin

## Abstract

The D76N variant of human β_2_-microglobulin (β_2_m) is the causative agent of a hereditary amyloid disease. Interestingly, D76N-associated amyloidosis has a distinctive pathology compared with aggregation of WT-β_2_m, which occurs in dialysis-related amyloidosis. A folding intermediate of WT-β_2_m, known as the I_T_-state, which contains a nonnative *trans* Pro-32, has been shown to be a key precursor of WT-β_2_m aggregation *in vitro*. However, how a single amino acid substitution enhances the rate of aggregation of D76N-β_2_m and gives rise to a different amyloid disease remained unclear. Using real-time refolding experiments monitored by CD and NMR, we show that the folding mechanisms of WT- and D76N-β_2_m are conserved in that both proteins fold slowly via an I_T_-state that has similar structural properties. Surprisingly, however, direct measurement of the equilibrium population of I_T_ using NMR showed no evidence for an increased population of the I_T_-state for D76N-β_2_m, ruling out previous models suggesting that this could explain its enhanced aggregation propensity. Producing a kinetically trapped analog of I_T_ by deleting the N-terminal six amino acids increases the aggregation rate of WT-β_2_m but slows aggregation of D76N-β_2_m, supporting the view that although the folding mechanisms of the two proteins are conserved, their aggregation mechanisms differ. The results exclude the I_T_-state as the origin of the rapid aggregation of D76N-β_2_m, suggesting that other nonnative states must cause its high aggregation rate. The results highlight how a single substitution at a solvent-exposed site can affect the mechanism of aggregation and the resulting disease.

β_2_-microglobulin (β_2_m) is a component of the major histocompatibility complex class 1 (MHC-1) which plays an important functional role in antigen presentation (1, 2). The MHC-1 complex consists of a monomeric heavy chain which is noncovalently assembled with a monomer of β_2_m during its biosynthesis in the endoplasmic reticulum (3). WT human β_2_m (WT-β_2_m) is a 99 residue, ∼12 kDa protein with a seven-stranded β-sandwich structure that is stabilized by a single disulfide bond between residues Cys-25 and Cys-80 (Fig. 1*a*) (4, 5). As part of its normal catabolic cycle, WT-β_2_m dissociates from the MHC-1 complex and is cleared from the serum via the kidneys (6). However, in individuals undergoing long-term hemodialysis for kidney failure, WT-β_2_m is not cleared effectively from the serum, resulting in an increase in its concentration from an average of 0.16 μm (5 healthy subjects) to 3.2 μm (11 patients) (6). The increased serum concentration contributes toward the formation of amyloid fibrils which typically deposit in collagen-rich joints, resulting in pathological bone and joint destruction in the disorder known as dialysis-related amyloidosis (6–8).

**Figure 1.**
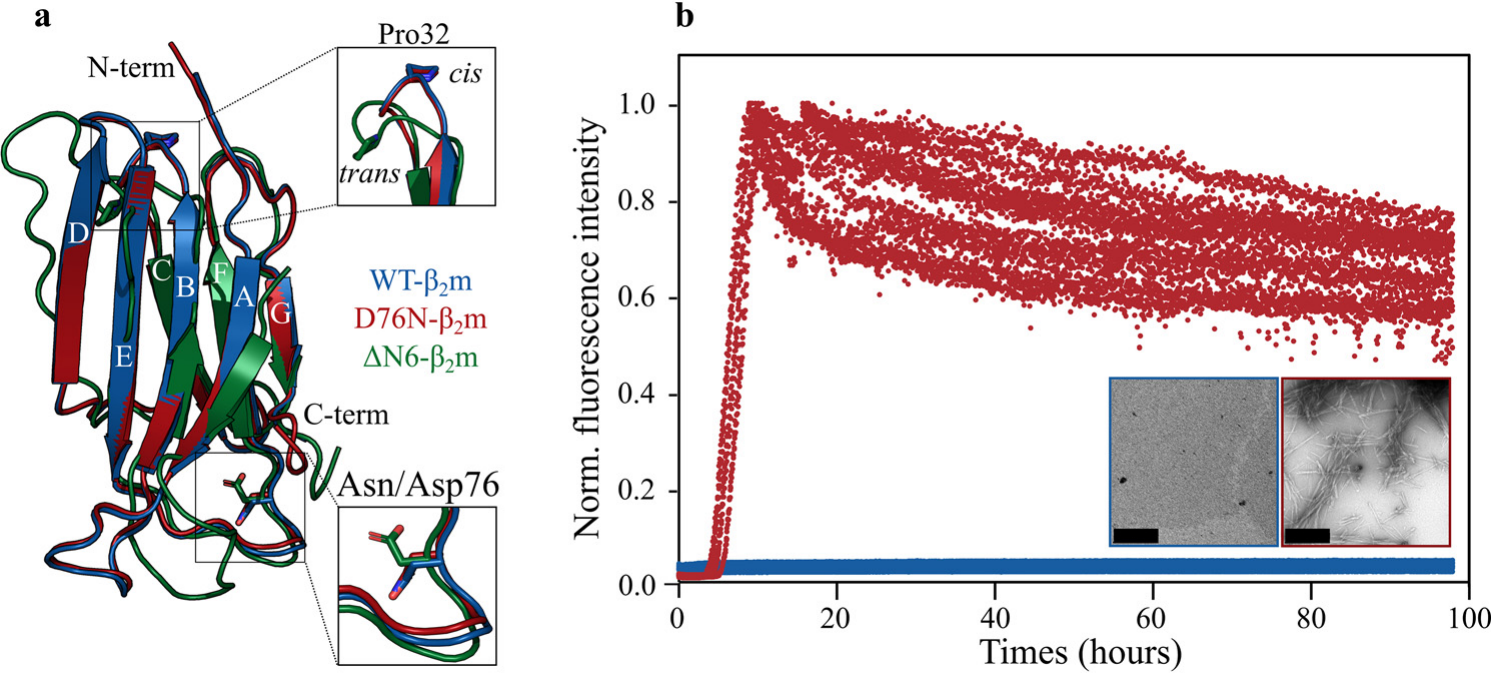
**Structure and amyloidogenicity of WT-, D76N-, and ΔN6-β_2_m.** *a*, superposition of the crystal structures of WT- β_2_m (*blue*) (PDB: 1LDS (51)) and D76N-β_2_m (*red*) (PDB: 4FXL (17)), and the lowest-energy structure of ΔN6-β_2_m determined using NMR (*green*) (PDB: 2XKU (16)). The *insets* highlight the BC loop, which contains Pro-32, and the EF-loop, which contains residue 76. *b*, aggregation kinetics of WT- and D76N-β_2_m (colored as in (*a*)) measured using ThT fluorescence. Experiments were performed with 30 μm protein in 25 mm sodium phosphate, pH 6.2, 137 mm NaCl, 10 μm ThT, 0.02% (w/v) NaN_3_, at 37°C, 600 rpm. 10 replicates are shown. Negative stain transmission EM images of the assay endpoints (taken after 100 h) are shown as *insets*, framed in the same colors. The *scale bar* corresponds to 300 nm.

The folding pathway of WT-β_2_m proceeds via a long-lived, structured, folding intermediate known as I_T_ (9–11). The slow rate of conversion from I_T_ to the native state (N-state) is caused by the necessary conversion of the peptidyl prolyl bond between His-31 and Pro-32 from a *trans* to *cis* configuration (11–13). Substitution of Pro-32 with natural or nonnatural amino acids has shown that the equilibrium population of the I_T_-state is directly proportional to the aggregation rate of WT-β_2_m (11–15). Consistent with this finding, a truncated form of WT-β_2_m in which the N-terminal six residues have been removed, enabling relaxation of Pro-32 from *cis* to *trans*, aggregates more rapidly than WT-β_2_m, presumably because this variant (known as ΔN6-β_2_m) cannot escape the I_T_-state (16). ΔN6-β_2_m is thus a structural mimic of the I_T_-state, and this is supported by the similarity of its ^1^H-^15^N-HSQC and far-UV CD spectra with those of the I_T_-state populated transiently during real-time refolding experiments (12, 16).

In 2012, the first naturally occurring β_2_m variant was identified in a French family as the causative agent of a hereditary, late-onset, fatal, and systemic amyloid disease (17). The amyloid fibrils that deposit in the visceral organs of these patients were shown to contain exclusively D76N-β_2_m, despite the individuals being heterozygous for the mutation and having normal renal function and normal serum β_2_m levels (0.11–0.13 μm) (17). Indeed, proteomic analysis of *ex vivo* amyloid fibrils from these patients failed to detect any WT- or ΔN6-β_2_m, nor was any truncated D76N-β_2_m detected (17). Moreover, no other common amyloid proteins were identified in these deposits by immunohistochemical staining. Most intriguingly, although WT-β_2_m does not aggregate *in vitro* at neutral pH, unless additives such as organic solvents, vigorous agitation, collagen, or glycosaminoglycans are included (18–20) or the protein is truncated at the N terminus (creating ΔN6-β_2_m) (21), D76N-β_2_m aggregates rapidly at neutral pH without the need of these interventions (17).

Various studies have been performed to try to rationalize the difference in the aggregation propensities of WT- and D76N-β_2_m. Because the I_T_-state is known to be critically important for WT-β_2_m aggregation, the folding pathway of D76N-β_2_m was investigated by Mangione *et al.* (22) using classical guanidine HCl–induced refolding/unfolding experiments, monitored by tryptophan fluorescence. These experiments suggested that D76N-β_2_m folds similarly to WT-β_2_m, with an initial rapid phase followed by a slow phase corresponding to the *trans* to *cis* isomerization of Pro-32 (22). Based on analysis of the kinetic data, the authors concluded that D76N-β_2_m populates the I_T_-state to ∼25% at equilibrium, in marked contrast with its population of only ∼5% for WT-β_2_m, rationalizing the increased amyloidogenicity of D76N-β_2_m (22). *In silico* studies have also suggested that the I_T_-state of D76N-β_2_m is structurally distinct from that of WT-β_2_m (23, 24), raising the possibility that these structural differences may also contribute to the enhanced aggregation propensity of D76N-β_2_m. Indeed, one such report suggested that the D76N-β_2_m I_T_-state has a larger solvent-exposed surface area, a more disordered D-strand and a greater solvation-free energy than the WT-β_2_m I_T_-state, all of which were proposed to contribute to the enhanced aggregation propensity of the protein (23). Alternative models (25) suggest instead that D76N-β_2_m forms two different I_T_-state structures: the first being the same as the WT-β_2_m I_T_-state and the second being unique to D76N-β_2_m by having unfolded N- and C-terminal regions. Interestingly, the second D76N-β_2_m I_T_-state was suggested to be more prone to oligomerization, its formation thus rationalizing the rapid aggregation of D76N-β_2_m (25).

To cast more light on the reasons for the enhanced amyloidogenicity of D76N-β_2_m, and specifically to distinguish between these different models, we analyzed the population and structure of the D76N-β_2_m I_T_-state directly, using real-time refolding experiments monitored by far-UV CD and heteronuclear NMR. These experiments provide direct structural and kinetic insights into the intermediate(s) formed during folding (26). The aggregation propensity of the D76N-β_2_m I_T_-state was also probed via the generation of an I_T_-state structural mimic at equilibrium by truncation of the N-terminal six amino acids of D76N-β_2_m (named ΔN6-D76N-β_2_m), inspired by the ΔN6-β_2_m variant (16). These results revealed that D76N-β_2_m folds through an I_T_-state that structurally mimics the I_T_-state of WT-β_2_m. Importantly, direct measurement of the population of the D76N-β_2_m I_T_-state at equilibrium using NMR revealed that this species is only rarely populated at equilibrium (the I_T_-state is below the detection threshold of ^1^H-^15^N-HSQC experiments at equilibrium) ruling out models that suggest an enhanced concentration of the I_T_-state as the rationale for the increased aggregation kinetics of D76N-β_2_m. Instead, we posit that the mutation of Asp to Asn, specifically at position 76 (27), alters the aggregation mechanism of β_2_m substantially, such that the rate of aggregation no longer depends on the structure or concentration of the I_T_ state.

## Results

### D76N-β_2_m folds via an I_T_-state that structurally resembles the I_T_-state of WT-β_2_m

**Figure 2.**
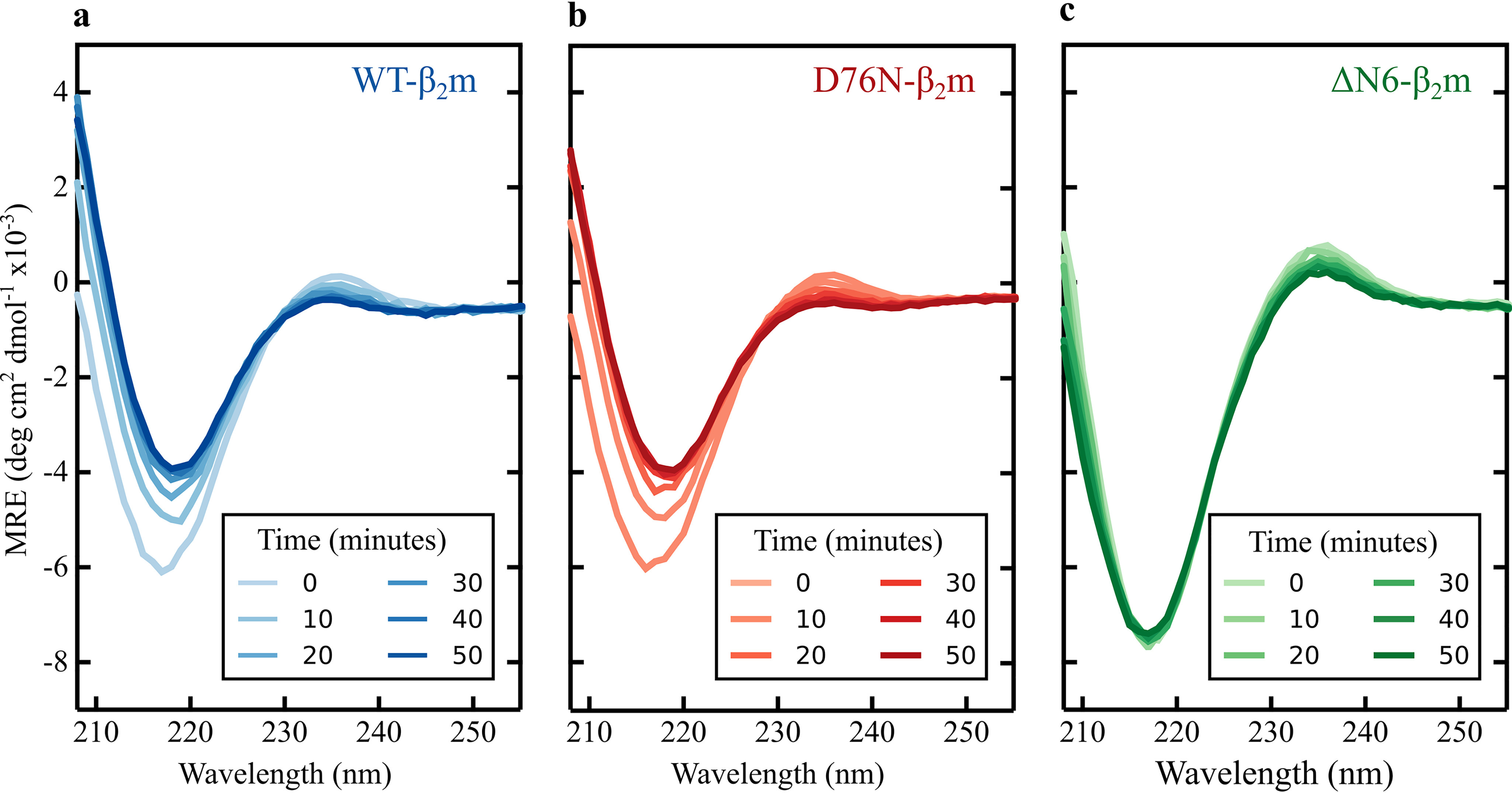
**Real-time refolding of WT-, D76N-, and ΔN6-β_2_m, monitored by far-UV CD.** *a*, WT-β_2_m is in blue. *b*, D76N-β_2_m is in red. *c*, ΔN6-β_2_m is in green. Spectra were recorded every minute over the refolding time course; however, only spectra acquired at 10-min intervals are shown here for clarity. In all plots, spectra are shaded darker as the time course progresses. These experiments were carried out at 20°C at a final protein concentration of 20 μm in 100 mm sodium phosphate buffer, pH 7.4. MRE corresponds to the molar ellipticity.

Despite sharing a common immunoglobulin fold and differing only in a single amino acid substitution at a solvent-exposed site (Fig. 1*a*), D76N-β_2_m aggregates rapidly at neutral pH, whereas WT-β_2_m does not aggregate into amyloid fibrils under the same conditions *in vitro* (Fig. 1*b*) (17). This raises the possibility that the difference in aggregation behavior of the two proteins could result from differences in (i) the population of a common amyloidogenic I_T_-state, (ii) the structural properties of the I_T_-state, or (iii) the proteins' aggregation mechanisms, such that D76N-β_2_m does not aggregate via the I_T_-state. To distinguish between these possibilities, we examined the conformational properties of the I_T_-states of WT- and D76N-β_2_m by real-time folding experiments monitored using far-UV CD and compared them with those of ΔN6-β_2_m. D76N- and WT-β_2_m have essentially identical native protein structures with a root-mean-square deviation (RMSD) of 0.3 Å (Fig. 1*a*) as well as identical far-UV CD spectra (Fig. S1). Despite an RMSD between ΔN6- and WT- or D76N-β_2_m of only 1.8 Å and 1.9 Å, respectively (Fig. 1*a*), the far-UV CD spectrum of ΔN6-β_2_m has a larger negative maximum at 216 nm than WT- or D76N-β_2_m (Fig. S1), presumably resulting from differences in the arrangement of aromatic side chains in the core of the proteins (14, 28). Analysis of the CD spectra of Pro-32 variants of β_2_m reported similar differences, and showed (assuming a two-state model) that the relative population of the I_T_- and N-states at equilibrium can be deduced directly from these spectra (14). Building on these results, WT- and D76N-β_2_m were each unfolded at acidic pH (see “Experimental procedures”). Folding was then initiated by rapidly increasing the pH to 7.4, and far-UV CD spectra were acquired as a function of time until folding was complete (Fig. 2, *a* and *b*). ΔN6-β_2_m, which is trapped at equilibrium in an I_T_-like state at pH 7.4, was similarly treated and included for comparison (Fig. 2*c*). The results showed, as expected (22), that both WT- and D76N-β_2_m fold rapidly (in less than a minute) to an I_T_-like state, yielding a far-UV CD spectrum with an intense negative maximum at 216 nm that is larger than that of their N-states and typical of that expected for a solution containing a significantly population of the I_T_-state (16, 22). Subsequent to this transition, slow refolding to the N-state occurs, which involves a decrease in signal intensity in the far-UV CD (Fig. 2, *a* and *b*). The refolding rate constant for this phase, which maps the I_T_- to N-state transition, was 1.03 × 10^−3^ ± 0.03 × 10^−3^ s^−1^ and 1.27 × 10^−3^ ± 0.03 × 10^−3^ s^−1^ for WT- and D76N-β_2_m, respectively, indicating that WT- and D76N-β_2_m fold to the N-state with similar rates (Fig. 2, *a* and *b*). Consistent with this interpretation, the slow phase is absent for ΔN6-β_2_m as this variant remains trapped in an I_T_-like state (Fig. 2*c*). These experiments confirm previous results which suggested that D76N-β_2_m folds slowly to its N-state via an I_T_-like species (16, 22) and reveal that this species resembles the I_T_-state of the WT protein, at least as judged by its far-UV CD spectrum.

**Figure 3.**
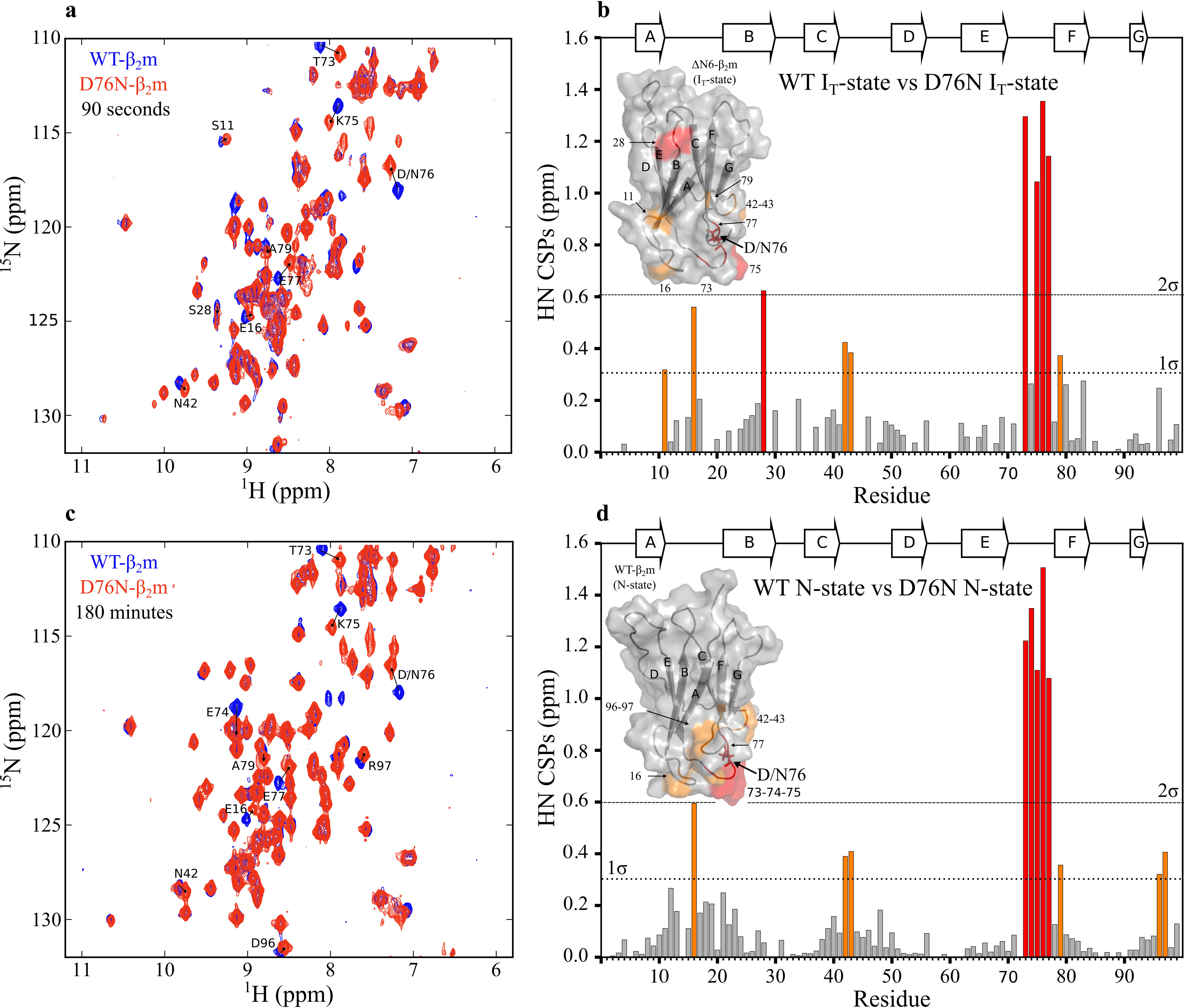
**Real-time refolding of WT- and D76N-β_2_m, monitored by ^1^H-^15^N NMR spectroscopy.** *a*, ^1^H-^15^N-SOFAST-HMQC spectra for WT-β_2_m (*blue*) and D76N-β_2_m (*red*) recorded 90 s after the initiation of refolding by pH jump (see “Experimental procedures”). Assignments of the 90-s spectra (I_T_-state) are shown in Figs. S2 and S4 for WT- and D76N-β_2_m, respectively. *b*, CSPs between spectra of WT- and D76N-β_2_m shown in (*a*). The CSP was calculated for the 58 peaks successfully assigned for the I_T_-state of both WT- and D76N-β_2_m (Figs. S2 and S4, respectively). *c*, ^1^H-^15^N-SOFAST-HMQC spectra of WT- β_2_m (*blue*) and D76N-β_2_m (*red*) recorded 180 min after the initiation of refolding. *d*, CSPs between spectra of WT- and D76N-β_2_m shown in (*c*). The CSP was calculated for the 87 peaks successfully assigned for the N-states of both WT- and D76N-β_2_m (Figs. S3 and S5, respectively). *a* and *c*, only positive contours are shown. ^1^H-^15^N resonances for Gly-18 and Gly-43 are therefore not present in this figure as they have negative intensities because of folding of the spectrum in the ^15^N dimension. Residues with significant chemical shift differences are labeled. *b* and *d*, CSPs <1σ (*dotted line*) from the mean of all CSPs are colored in *gray*; those between 1σ and 2σ are colored *orange*, and > 2σ from the mean are colored *red*. CSPs are mapped onto the solution structures of ΔN6- (PDB: 2XKU (16)) or WT-β_2_m (PDB: 2XKS (16)) for (*b*) and (*d*), respectively, using the same color code. These experiments were carried out at 20°C at a final protein concentration of 300 μm in 1.0 M urea and 167 mm sodium phosphate buffer, pH 7.4.

To obtain more detailed information about the structural properties of the D76N-β_2_m I_T_-state, refolding was also monitored in real-time using NMR. We first obtained a full backbone resonance assignment for native D76N-β_2_m, as this information was not available in the Biological Magnetic Resonance Data Bank (BMRB) (see “Experimental procedures”). Refolding experiments were initiated by rapidly increasing the pH of the acid-unfolded proteins to pH 7.4. The first ^1^H-^15^N-SOFAST-HMQC spectra of WT- and D76N-β_2_m obtained 90 s after the initiation of refolding show well-dispersed peaks, consistent with the presence of the structured I_T_-state, which is expected to dominate the refolding reaction at this time point (Fig. 3*a* and Figs. S2 and S4). It is interesting to note that ∼14 and ∼13% of the species populated at this time correspond to native WT- and D76N-β_2_m, respectively, as judged by the intensity of resonances unique to the N-state in each spectrum. Importantly, these spectra are distinct from those of the earlier intermediate of WT-β_2_m (I_1_) and murine β_2_m observed previously using nonuniform sampling NMR methods, which gives rise to very broad spectra and species shown not to be amyloidogenic (29). As expected, the spectra of the I_T_-states of WT- and D76N-β_2_m are very similar to one another (Fig. 3*a*), as well as to spectra previously observed for the WT-β_2_m I_T_-state (22) and ΔN6-β_2_m (16) (obtained under similar conditions, with identical pH and salt concentrations). Using the previously assigned ΔN6-, WT-, and D76N-β_2_m spectra in combination, amino acid assignments were transferred to the spectra acquired after a 90-s refolding time (Figs. S2 and S4). 69 peaks were successfully assigned for WT-β_2_m and 58 for D76N-β_2_m, allowing the chemical shifts of resonances in the I_T_-states of WT- and D76N-β_2_m to be compared (Fig. 3*b*). This showed that significant chemical shift perturbations (CSPs) are observed only for residues in the EF-loop (residues 71 to 78, which contains the D76N substitution) and the structurally adjacent AB-loop (residues 12 to 20) (Fig. 1*a* and Fig. 3*b*). The ^1^H-^15^N-SOFAST-HMQC spectra of native (N-state) WT- and D76N-β_2_m (obtained after a folding time of 180 min (Fig. 3*c*, Figs. S3 and S4) are also similar, with the only significant chemical shift differences again involving residues in the AB- and EF-loops (Fig. 3*d*). The similar CSPs between WT- and D76N-β_2_m at 90 s (I_T_-state) and 180 min (N-state) refolding times (Fig. 3, *b* and *d*) show that the folding of both proteins involves a kinetically long-lived I_T_-state that has similar structural properties for both β_2_m variants, at least as judged by these approaches.

**Figure 4.**
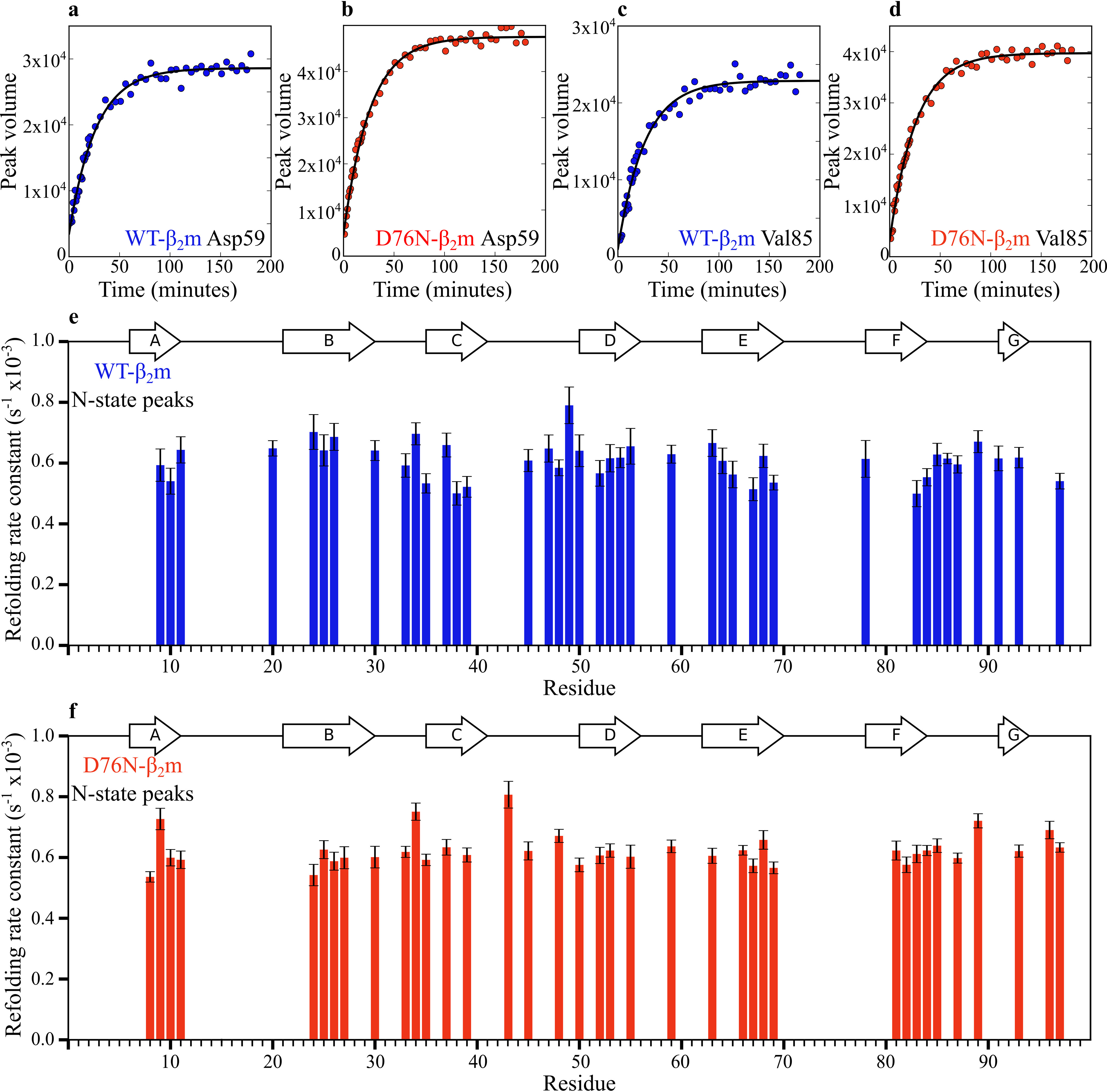
**Single-residue refolding rates for N-state peaks of WT- and D76N-β_2_m, monitored by NMR spectroscopy.** *a*–*d*, representative data and fits in *black* for single residue folding rates fitted with Equation 1 (see “Experimental procedures”). *e* and *f*, the rate constants for individual residues that could be measured with confidence (where the error on the fit is no more than three median absolute deviations of all errors within each data set) are shown in (*e*) and (*f*) for WT- and D76N-β_2_m, respectively. *Error bars* are the fitting errors.

### The β_2_m folding energy landscape is unperturbed by the D76N substitution

Given the similarities in the folding mechanisms of WT- and D76N-β_2_m, the remarkable difference in their rates of aggregation into amyloid could result from differences in the population of the I_T_-state at equilibrium, which would be reflected by differences in the rate of folding/unfolding of I_T_-state to/from the N-state. Indeed, such a scenario was posited previously based on analysis of their folding kinetics using tryptophan fluorescence (22). Consistent with this view, the population of the I_T_-state in WT-β_2_m variants (such as P32G-, P5G-, and ΔN6-β_2_m) have been shown to correlate with their aggregation rates (14). The rate of the I_T_- to N-state transition of WT- and D76N-β_2_m was investigated at the single residue level by fitting the ^1^H-^15^N-SOFAST-HMQC peak volumes for resonances which have a unique chemical shift in their N-states (*i.e.* they do not overlap with peaks arising from I_T_-state). For WT-β_2_m/D76N-β_2_m 70/66 peaks could be identified as unique to their N-states (Figs. S3 and S5). The intensity of these peaks was monitored as a function of the refolding time and fitted to a single exponential function (see “Experimental procedures”) (Fig. 4, *a*–*d*), from which 40/37 per residue refolding rate constants, respectively, could be determined with confidence (Fig. 4, *e* and *f*). Of note is that the peak volume is not zero in the initial spectrum obtained after 90 s, reflecting a small population (<15%) of molecules that fold rapidly to the N-state presumably because they represent the small population of molecules with a *cis* Pro-32 in the unfolded state (Fig. 4, *a*–*d*). The data revealed that the I_T_- to N-state transition proceeds at a similar rate for all residues monitored for WT- and D76N-β_2_m, with median rate constants of 0.62 × 10^−3^ ± 0.05 × 10^−3^ s^−1^ and 0.63 × 10^−3^ ± 0.04 × 10^−3^ s^−1^, respectively. Hence the energy barrier for the I_T_- to N-state transition is similar for both proteins.

**Figure 5.**
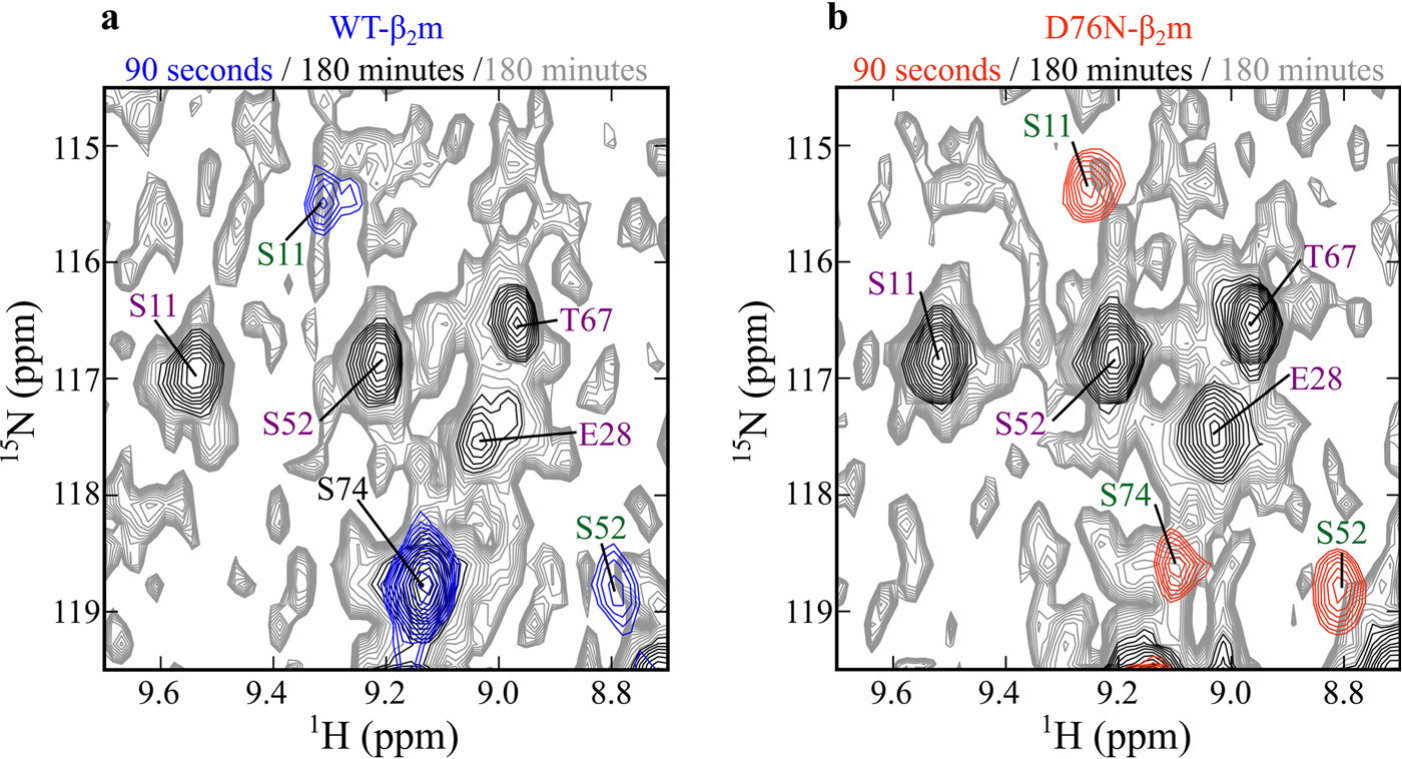
**Searching for I_T_-state peaks in the N-state ^1^H-^15^N-SOFAST-HMQC spectra of native WT- and D76N-β_2_m.** *a* and *b*, the spectra of (*a*) WT- and (*b*) D76N-β_2_m taken at 180 min (*black* contours) after refolding are compared with the corresponding spectra taken at 90 s (*blue* contours for WT-β_2_m and *red* contours for D76N-β_2_m). The native protein spectra obtained after a refolding time of 180 min are contoured to show the spectral noise (*gray*) down to a level 12-fold below that of the lowest *black* contour. The peaks unique to the I_T_-state are labeled in *green*, those unique to the N-state are labeled in *purple*, and peaks common between the I_T_-state and the N-state are labeled in *black*. There is no evidence of observable resonances from the I_T_-state in the spectra of the native proteins, consistent with a very low population of I_T_ at equilibrium.

A similar kinetic analysis was carried out focusing on peaks which are unique to the I_T_-state (*i.e.* they do not overlap with peaks rising from N-state) (Fig. S6). In the spectra obtained after a 90-s refolding time, 26/25 peaks are unique to the I_T_-states for WT-β_2_m/D76N-β_2_m, respectively (Figs. S2 and S4). The intensity of these peaks was also monitored as a function of the refolding time and fitted to a single exponential (see “Experimental procedures”) (Fig. S6, *a*–*d*), from which 20/19 per residue refolding rate constants for WT- and D76N-β_2_m, respectively, could be determined with confidence (Fig. S6, *e* and *f*). This analysis also showed similar kinetic behavior for WT- and D76N-β_2_m, with the decrease in intensity of I_T_-state peaks occurring with median rate constants of 0.56 × 10^−3^ s^−1^ ± 0.06 × 10^−3^ s^−1^ and 0.49 × 10^−3^ ± 0.05 × 10^−3^ s^−1^, respectively (Fig. S6, *e* and *f*). The similarity in rate constants for different residues throughout the protein sequence for these transitions provides strong evidence in support of a two-state I_T_- to N-state transition. In addition, the results show that the energy barrier between the I_T_-state and the N-state is essentially unperturbed by the D76N substitution.

**Figure 6.**
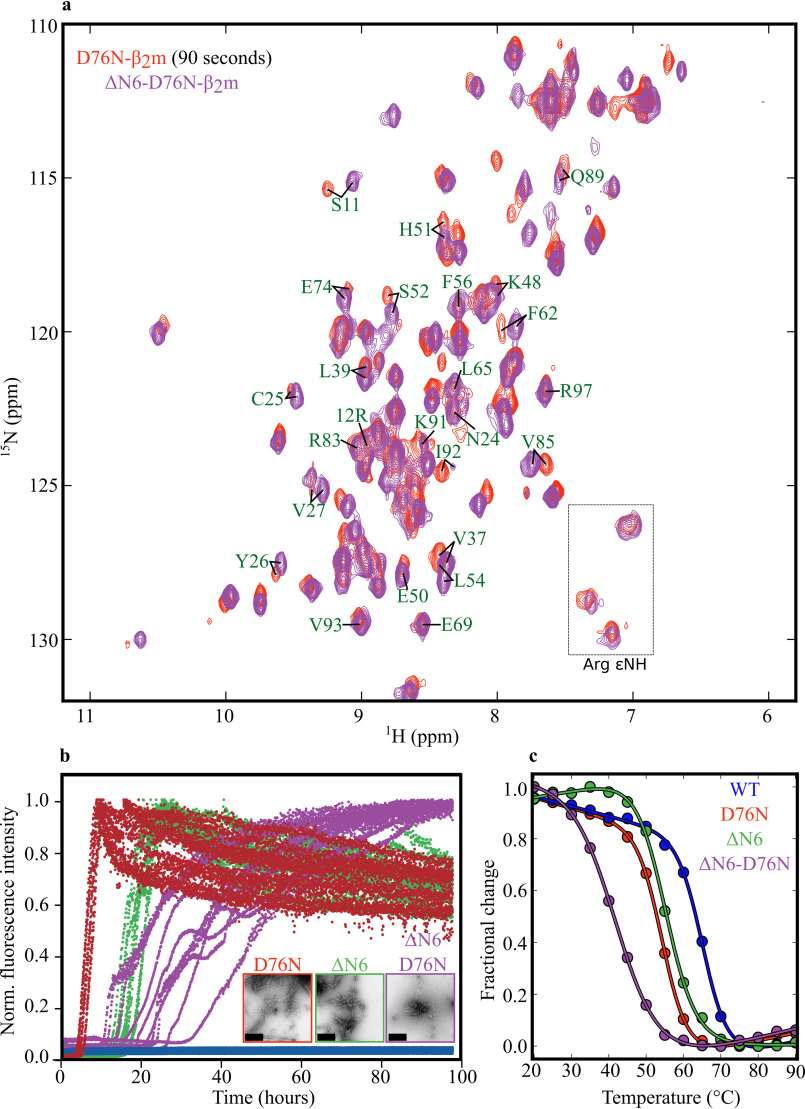
**Characterization of ΔN6-D76N-β_2_m.** *a*, superposition of the ^1^H-^15^N-SOFAST-HMQC spectra of ΔN6-D76N β_2_m (*purple*) (80 μm protein in 25 mm sodium phosphate pH 7.4, 20°C) and D76N-β_2_m after 90 s refolding time (*red*). The 25 peaks unique to the I_T_-state are labeled in *green*. *b*, Aggregation of ΔN6-D76N-β_2_m (*purple*), D76N-β_2_m (*red*), ΔN6-β_2_m (*green*), and WT-β_2_m (*blue*) (30 μm protein in 25 mm sodium phosphate pH 6.2, 137 mm NaCl, 10 μm ThT, 0.02% (w/v) NaN_3_, 37°C, 600 rpm). Negative stain transmission EM images of amyloid fibrils from reaction end point (taken after 100 h) are shown alongside, framed in the same colors. The *scale bar* corresponds to 200 nm. Please note that the ThT curves and EM image for D76N-β_2_m and WT-β_2_m are reproduced from Fig. 1*b* to allow direct comparison with the other proteins shown. *c*, stability of different β_2_m variants monitored by far-UV CD at 216 nm. The data were fitted using an equation describing a two-state exchange model using the CDPal software package (47) for calculation of *T_m,app_* values (see “Experimental procedures”). The temperature ramp experiment was carried out in 25 mm sodium phosphate, pH 6.2, in the range 20–90°C in 5°C steps.

The unique chemical shifts for residues in the I_T_- and N-states also enable the equilibrium populations of the I_T_- and N-states in WT- and D76N-β_2_m to be directly determined using NMR. Previous results have shown that the population of the I_T_-state is less than 5% for WT-β_2_m at equilibrium under the conditions used (22). Consistent with this, despite contouring into the noise of the native WT-β_2_m spectrum, resonances unique to the I_T_-state could not be observed. The lowest contour level of this spectrum is 12-fold below that of the other spectra shown (Fig. 5*a*). Despite high signal-to-noise of these spectra, no evidence for resonances which are unique to the I_T_-state of WT-β_2_m could be observed in the noise of its N-state spectrum (see for example resonances for Ser-11 and Ser-52 in Fig. 5*a*), implying a low equilibrium population of the I_T_-state. Importantly, the lack of detectable I_T_-state resonances in the spectrum of native D76N-β_2_m (Fig. 5*b*) demonstrates a similar low equilibrium population of I_T_-state for this protein, consistent with the similarities of these variants' far-UV CD spectra presented in Fig. S1. Thus, the D76N-β_2_m I_T_-state has similar structure, relative population and interconversion rates with the N-state as the WT-β_2_m I_T_-state, providing clear evidence that the amyloidogenicity of D76N-β_2_m cannot be attributed to differences in the I_T_-state.

### Generation of a kinetically trapped D76N-β_2_m I_T_-state mimic

To determine the aggregation propensity of the D76N-β_2_m I_T_-state directly, a truncated product of the D76N-β_2_m variant was produced in which the N-terminal six residues were removed, inspired by previous findings that ΔN6-β_2_m mimics the I_T_-state of WT-β_2_m (16, 29, 30), referred to as ΔN6-D76N-β_2_m (see “Experimental procedures”). As anticipated, the ^1^H-^15^N-SOFAST-HMQC spectrum of this variant closely resembles the spectrum of the D76N-β_2_m I_T_-state captured transiently during refolding, indicating that ΔN6-D76N-β_2_m is indeed an I_T_-state mimic of D76N-β_2_m (Fig. 6*a*). Interestingly, measurement of the rate of aggregation of the different proteins into amyloid fibrils using ThT fluorescence showed that ΔN6-D76N-β_2_m is *less* aggregation-prone than its full-length counterpart, by contrast with truncation of the N-terminal six residues from WT-β_2_m which dramatically *increases* the rate of its aggregation (compare Fig. 1*b* and Fig. 6*b*). Fitting the normalized ThT fluorescence intensity data yielded aggregation half-time (*t*_half_) values of 24.7 ± 9.5 h for ΔN6-D76N-β_2_m, 18.0 ± 1.9 h for ΔN6-β_2_m, and 6.6 ± 0.7 h for D76N-β_2_m (Table 1). Hence, of these three variants, D76N-β_2_m aggregates most rapidly despite containing a *cis* Pro-32 and an intact N-terminal sequence.

**Table 1:** **Aggregation rates and protein stability of WT-β_2_m and the three variants (D76N-, ΔN6-, and ΔN6-D76N-β_2_m)** Measurements were made in 30 μm protein in 25 mm sodium phosphate, pH 6.2, 137 mm NaCl, 10 μm ThT, 0.02% (w/v) NaN_3_, 37°C, 600 rpm for the *t*_half_, and 30 μm protein in 25 mm sodium phosphate, pH 6.2 for the *T_m,app_*. Note that WT-β_2_m does not aggregate under the conditions used here over the time span measured.

Variant	Aggregation *t*_half_ (h)	*T_m,app_* (°C)
WT-β_2_m	—	65.5 ± 0.5
D76N-β_2_m	6.6 ± 0.7	54.6 ± 0.1
ΔN6-β_2_m	18.0 ± 1.9	55.2 ± 0.5
ΔN6-D76N-β_2_m	24.7 ± 9.5	42.8 ± 0.9

Finally, the effect of deleting the N-terminal six residues on the stability of D76N-β_2_m was measured using temperature denaturation monitored by far-UV CD (Fig. 6*c*). The results revealed an apparent midpoint temperature (*T_m,app_*) of denaturation (Table 1) with the rank order of stability ΔN6-D76N-β_2_m < D76N-β_2_m ∼ ΔN6-β_2_m < WT-β_2_m. The results demonstrate that the *t*_half_ aggregation does not correlate with thermodynamic stability. Interestingly, the results also showed that the difference in *T_m,app_* between ΔN6- and WT-β_2_m is 10.3 °C, a value similar to that obtained by deletion of the N-terminal six residues in D76N-β_2_m (11.8 °C) (Table 1). Thus, there is little cross-talk between the N-terminal hexapeptide and the effect of the amino acid substitution at position 76 on protein stability.

## Discussion

The native-like folding intermediate of WT-β_2_m, known as the I_T_-state, is central to the mechanism of its assembly into amyloid (14, 31). Here, we have examined in detail the contribution of the I_T_-state to the aggregation mechanism of the closely related D76N-β_2_m variant, building on previous results which suggested that the population and/or structural properties of this state could rationalize the dramatically enhanced ability of the protein to aggregate into amyloid both *in vitro* and *in vivo* (22). The slow folding rate of WT- and D76N-β_2_m was exploited here to enable direct analysis of the I_T_- to N-state transition using real-time far-UV CD and NMR spectroscopy. The results revealed that D76N-β_2_m folds via an I_T_-state which structurally resembles the WT-β_2_m I_T_-state. Analysis of the refolding kinetics in residue-specific detail showed that the activation barrier between the I_T_- and N-states in WT- and D76N-β_2_m is similar. This implies a similar degree of destabilization of the I_T_-state, transition state and N-state by the substitution of Asp to Asn at position 76 (in agreement with all species having native-like structural properties). Moreover, the relative populations of the I_T_- and N-state at equilibrium are also not perturbed by the D76N substitution. Hence, by contrast with previous reports (22), our evidence shows that the enhanced amyloidogenicity of D76N-β_2_m cannot be explained by increased population of I_T_-state or by any substantial differences in its structural properties (although subtle differences in conformation not reflected in ^1^H/^15^N chemical shifts cannot be ruled out). Finally, the decreased stability of D76N-β_2_m relative to the WT protein does not explain its increased amyloid potential, because other β_2_m variants with similar or even further reduced stability compared with D76N-β_2_m, including murine-β_2_m (29), V37A-β_2_m (32), and the ΔN6-D76N-β_2_m variant described here, all aggregate more slowly than D76N-β_2_m.

The similarity of the WT- and D76N-β_2_m I_T_-states is further suggested by the similarity in the aggregation rates of ΔN6-β_2_m and ΔN6-D76N-β_2_m, both of which are presumably trapped in an I_T_-like state. Strikingly, this rate is slower than that of the parent D76N-β_2_m variant, demonstrating that although the D76N-β_2_m I_T_-state is aggregation-prone, its formation cannot be rate-determining for aggregation of the full-length protein. This suggests that D76N-β_2_m aggregates by a mechanism distinct from that of its WT counterpart for which the I_T_-state population determines the rate of aggregation (11). Instead aggregation of D76N-β_2_m could be initiated by formation of a different nonnative but structured species, possibly the previously identified N*-state observed in D76N-β_2_m crystals (33). Alternatively, aggregation may occur from more highly disordered state(s) of the protein, with the D76N substitution increasing the amyloidogenicity of these species by altering their conformational properties. Such a mechanism has been posited for immunoglobulin light chains associated with light chain amyloidosis based on the orientation of the two β-strands linked by the disulfide bond in the native monomer and in the amyloid fold (34). In addition, the role of flanking regions in tailoring amyloidogenicity has been observed in several other proteins that aggregate from a disordered state, including α-synuclein (35) and tau (36). A different aggregation pathway and precursor species in D76N-β_2_m could also explain the subtle differences in the WT- and D76N-β_2_m fibril secondary structures determined using solid state NMR and/or cryo-EM (33, 37–40).

In summary, the results presented here demonstrate that the mechanisms of aggregation of WT- and D76N-β_2_m differ significantly, with the WT protein aggregating via formation of the I_T_-state, whereas for D76N-β_2_m a different native-like-state (N*-state) (33) or perhaps a more highly unfolded state (41) could be rate-determining for aggregation. These differences in mechanism, involving different precursor(s), may also explain the radical differences between the systemic amyloidosis caused by D76N-β_2_m and the pathology of dialysis-related amyloidosis caused by the WT protein. Indeed, at normal serum concentrations, D76N-β_2_m aggregates into amyloid without involvement of the WT protein in these heterozygous individuals (22). By contrast, for WT-β_2_m aggregation involves truncation of the N terminus to form ΔN6-β_2_m, the isomerization of *cis* Pro-32 to *trans* (29, 30), and the involvement of collagen, glycosaminoglycans, and other extracellular factors to create amyloid that deposits specifically in the joints (18, 32, 42, 43). Our results thus highlight the fundamental difference in the *in vitro* aggregation mechanism and the consequences in diseases brought by a single amino acid substitution in a solvent-exposed loop of a protein with a simple 99-residue immunoglobulin fold.

## Experimental procedures

### Protein expression and purification

^14^N-, ^15^N-, and ^15^N-^13^C-labeled proteins were expressed and purified as described previously (30). D76N-ΔN6-β_2_m was particularly prone to precipitation when resuspending the lyophilized material during purification, and so care was taken to ensure that resuspension was always carried out in 20 mm sodium phosphate, pH 7.4. All proteins were purified in the last step using gel filtration and care was taken to only collect the center of the monomer peak so as to exclude the possibility of oligomers in the preparations. Analysis using SEC-MALLS, native electrospray ionization–MS and by re-injecting the protein onto the column after concentration did not reveal the detectable presence of oligomers in the preparations.

### Real-time refolding monitored by far-UV CD

Proteins (30 μm) were dialyzed against the unfolding solution (0.8 m urea, 25 mm sodium phosphate buffer at pH 2.5) for 1 h. To initiate refolding, the unfolded proteins were rapidly diluted with 300 mm sodium phosphate buffer, pH 7.4 (2:1 (v/v) unfolded protein:refolding buffer) at 20°C. Data acquisition was initiated immediately after addition of the refolding buffer into the CD cuvette which already contained the unfolded protein (dead-time ∼1 s). Spectra (200–260 nm) were acquired using a Chirascan^TM^ Plus CD spectrometer (Applied Photophysics). One spectrum was recorded per minute using a step size of 1 nm and a sampling time of 0.5 s per point.

### Real-time refolding monitored using NMR

Protein samples (450 μm) were dialyzed against the unfolding solution (1.5 m urea, 25 mm sodium phosphate buffer at pH 2.5 containing 10% (v/v) D_2_O) for 1 h. To initiate refolding, 150 μl of refolding buffer (500 mm sodium phosphate, pH 7.4) was added to 350 μl of each unfolded protein (final protein concentration 300 μm in 167 mm sodium phosphate buffer, pH 7.4). These experiments were carried out at 20°C. The sample was immediately added to the NMR tube and data acquisition was initiated (dead-time ∼30 s). The folding reaction was monitored by acquiring ^1^H-^15^N-SOFAST-HMQC (44) spectra every 60 s, with 100 points in f1 (^15^N) and 956 in f2 (^1^H), and two scans were acquired per increment. Spectra were recorded on a 600 MHz Bruker AVANCE III HD spectrometer equipped with a 5 mm QCI-P (proton-observe inverse quadruple resonance) cryoprobe, using spectral widths of 15.97 ppm in f2 and 22.00 ppm in f1.

Spectra were processed in NMRPipe (45) and analyzed with the software package PINT (46). Peak volumes were determined by fitting to a Lorentzian line shape. The total peak volume of each residue was plotted as a function of time and fitted to a single exponential to determine the refolding rate constant:

(Eq. 1) $$y=-ae^{-bx}+c$$

or

(Eq. 2) $$y=ae^{-bx}+c$$

where y is the intensity of the chosen peak at time x, c is the value of y at infinite time, a is the initial intensity, and b is the rate constant. For positive peaks, Equation 1 was used to fit peaks unique to the N-state and Equation 2 was used to fit peaks unique to the I_T_-state.

CSPs were calculated using Equation 3:

(Eq. 3) CSP= 5δH12 + δN152

where δH1 and δN15 are the differences in the ^1^H and ^15^N chemical shifts for the two resonances being compared.

### Thermal denaturation monitored by far-UV CD

For thermal denaturation experiments an initial spectrum of the sample (20 μm protein in 25 mm sodium phosphate buffer, pH 7.4), was obtained at 25°C. The temperature of the solution was decreased to 20°C, and then increased in 5°C steps with an equilibration time of 120 s at each temperature, up to a final temperature of 90°C. At the end of the temperature ramp, the sample was cooled to 25°C and a spectrum acquired to determine whether the transition was reversible. Each spectrum was acquired from 190 nm to 260 nm with a step size of 1 nm and 1 s per point sampling. Two spectra were acquired for each temperature and averaged. The path length used was 1 mm. The data were fitted to a two-state equilibrium (Equation 4) using the software package CDPal (47).

(Eq. 4) $$E=\;e^{-\;\frac{\Delta H_{m}}{R}\left(\frac{1}{T_{m}}\;-\;\frac{1}{T}\right)\;-\;\frac{\Delta C_{p}}{R}\left(\frac{T_{m}}{T}-1\;+\;\ln\;\left(\frac{T}{T_{m}}\right)\right)}$$

Where $\Delta H_{m}$ is the change in enthalpy at the denaturation midpoint $T_{m}$, $\Delta C_{p}$ is the difference in heat capacity between the two states, *R* is the gas constant and *T* the temperature (Kelvin). $\Delta C_{p}$ was assumed to be independent of temperature. Because the thermal denaturation process was not fully reversible, *T_m,app_* values are quoted.

### In vitro fibrillation assays

Protein samples (stored either as lyophilized powder and resolubilized immediately before use in 25 mm sodium phosphate buffer pH 7.4, or as concentrated solution at −80°C) were centrifuged at 14,000 × *g* for 10 min, the supernatant was filtered (0.22 μm, Millipore), diluting the same as appropriate to give a final protein concentration of 30 μm in 25 mm sodium phosphate pH 6.2, 137 mm NaCl, 10 μm ThT, 0.02% (w/v) NaN_3_. Each protein (10 replicates, 100 μl each) was added to Corning 96-well polystyrene microtiter plates, sealed with clear polyolefin film (STARLAB) and incubated at 37°C for at least 48 h with constant shaking at 600 revolutions per minute (rpm). ThT fluorescence was monitored (excitation 440 nm and emission 480 nm) with a Fluostar Optima, BMG Labtech plate reader.

*t*_half_ values were calculated by fitting normalized data (between 0 to 1) for each replicate to Equation 5 and determining the time taken to reach half the maximal intensity:

(Eq. 5) $$Y\left(t\right)=A+\frac{K-A}{\left(\begin{array}{c} 1+Qe^{-B\left(\begin{array}{c} t-M \end{array}\right)\frac{1}{v}} \end{array}\right)}$$

where A is the pretransition baseline (lower asymptote), K is the posttransition baseline (upper asymptote), B is the growth rate, and M is the time of maximal growth. Q and v are parameters which affect the transitions from and to the growth phase, Y is the normalized signal, and t is time (48, 49).

### Negative stain transmission EM

Carbon-coated copper EM grids were placed coated-side down onto sample drops containing undiluted material from the *in vitro* fibrillation assay for 30 s. The grids were then blotted with filter paper to remove excess solvent and sample. Grids were then placed onto drops of 2% (w/v) uranyl acetate for 30 s, blotted again, and air-dried. Images were taken using a Jeol 1400 microscope using a 120 keV laboratory filament and Gatan US1000XP 2k × 2k CCD camera.

### D76N-β_2_m NMR assignment

The assignment of D76N-β_2_m was performed in 25 mm sodium phosphate, 83 mm sodium chloride at pH 7.4 and 25°C. ^15^N and ^15^C uniformly labeled protein was used to acquire all NMR experiments needed to accomplish the backbone and side chains assignment. Triple resonance HNCO, HNCA, HN(co)CA, HN(co)CACB, (h)CCH-TOCSY, H(c)CH-TOCSY NMR experiments were recorded on a Bruker AVANCE III HD 750 MHz spectrometer equipped with triple resonance inverse cryoprobe. Spectra were processed using NMRPipe and analyzed using CcpNmr Analysis (version 2.4) (50).

## Data Availability

All raw data from the results presented will be made available upon request. Please contact Sheena Radford (s.e.radford@leeds.ac.uk). ^1^H, ^15^N, and ^13^C chemical shift assignments for D76N were deposited in the Biological Magnetic Resonance Data Bank (accession number 50302).

## Supplementary Material

Supporting Information
