# Supplementary material for: The role of the IT-state in D76N β2-microglobulin amyloid assembly: A crucial intermediate or an innocuous bystander?
Source: J Biol Chem. 2020 Jul 13;295(35):12474–84. doi: 10.1074/jbc.RA120.014901 (PMC7458819; doi:10.1074/jbc.RA120.014901)
Supplement: Supporting Information [file supp_295_35_12474__index.html]

The role of the IT-state in D76N β2-microglobulin amyloid assembly: a crucial intermediate or an innocuous bystander? — The role of the IT-state in D76N-β2m aggregation — The role of the IT-state in D76N β2-microglobulin amyloid assembly: A crucial intermediate or an innocuous bystander? — The role of the IT-state in D76N-β2m aggregation — Supporting Information 

# The role of the IT-state in D76N β2-microglobulin amyloid assembly: A crucial intermediate or an innocuous bystander?

## Supporting Information

- Supporting Information (to be published online) - Supporting information to be published
