# Supplementary material for: The role of the IT-state in D76N β2-microglobulin amyloid assembly: A crucial intermediate or an innocuous bystander?
Source: J Biol Chem. 2020 Jul 13;295(35):12474–84. doi: 10.1074/jbc.RA120.014901 (PMC7458819; doi:10.1074/jbc.RA120.014901)
Supplement: Supporting Information [file supp_RA120.014901_161646_1_supp_560308_qd1ry1.pdf]

Supporting Information: The role of the I_T-state in D76N β_2m amyloid assembly a crucial intermediate or an innocuous bystander?

Hugh I. Smith[1$], Nicolas Guthertz[1$], Emma E. Cawood[1,2], Roberto Maya-Martinez[1], Alexander L. Breeze[1], Sheena E. Radford[1]

From the [1]Astbury Centre for Structural Molecular Biology, School of Molecular & Cellular Biology, Faculty of Biological Sciences, University of Leeds, Leeds, LS2 9JT, UK and [2]School of Chemistry, University of Leeds, Leeds LS2 9JT, UK

Running title: Supplementary Information: The role of the I_T-state in D76N β_2m aggregation

*To whom correspondence should be addressed: Sheena E. Radford, Astbury Centre for Structural Molecular Biology and School of Molecular and Cellular Biology, University of Leeds, Leeds LS2 9JT Telephone: +44 113 343 3170; email: s.e.radford@leeds.ac.uk.

[$]Equal contribution

Supplementary Figure S1

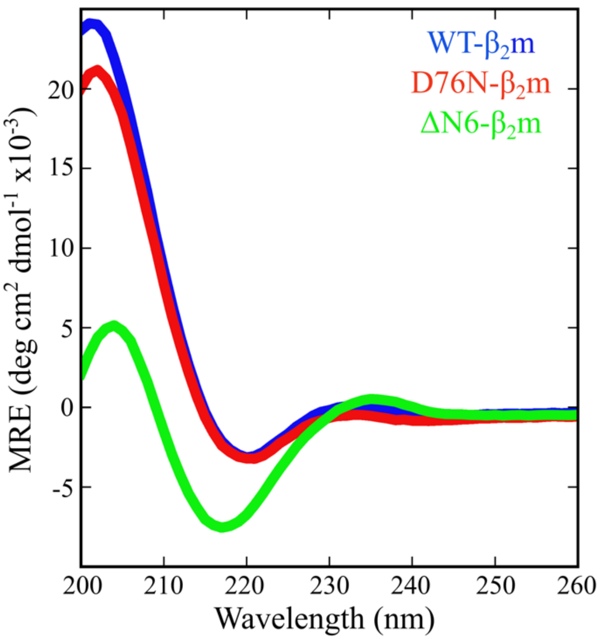

Supplementary Fig. S1. Comparison of the far-UV CD spectra of WT-, D76N- and ΔN6-β_2m. WT-β_2m is in blue, D76N-β_2m is in red and ΔN6-β_2m is in green. The experimental conditions are 20 μM protein, 25 mM sodium phosphate pH 7.4 at 25 °C.

Supplementary Figure S2

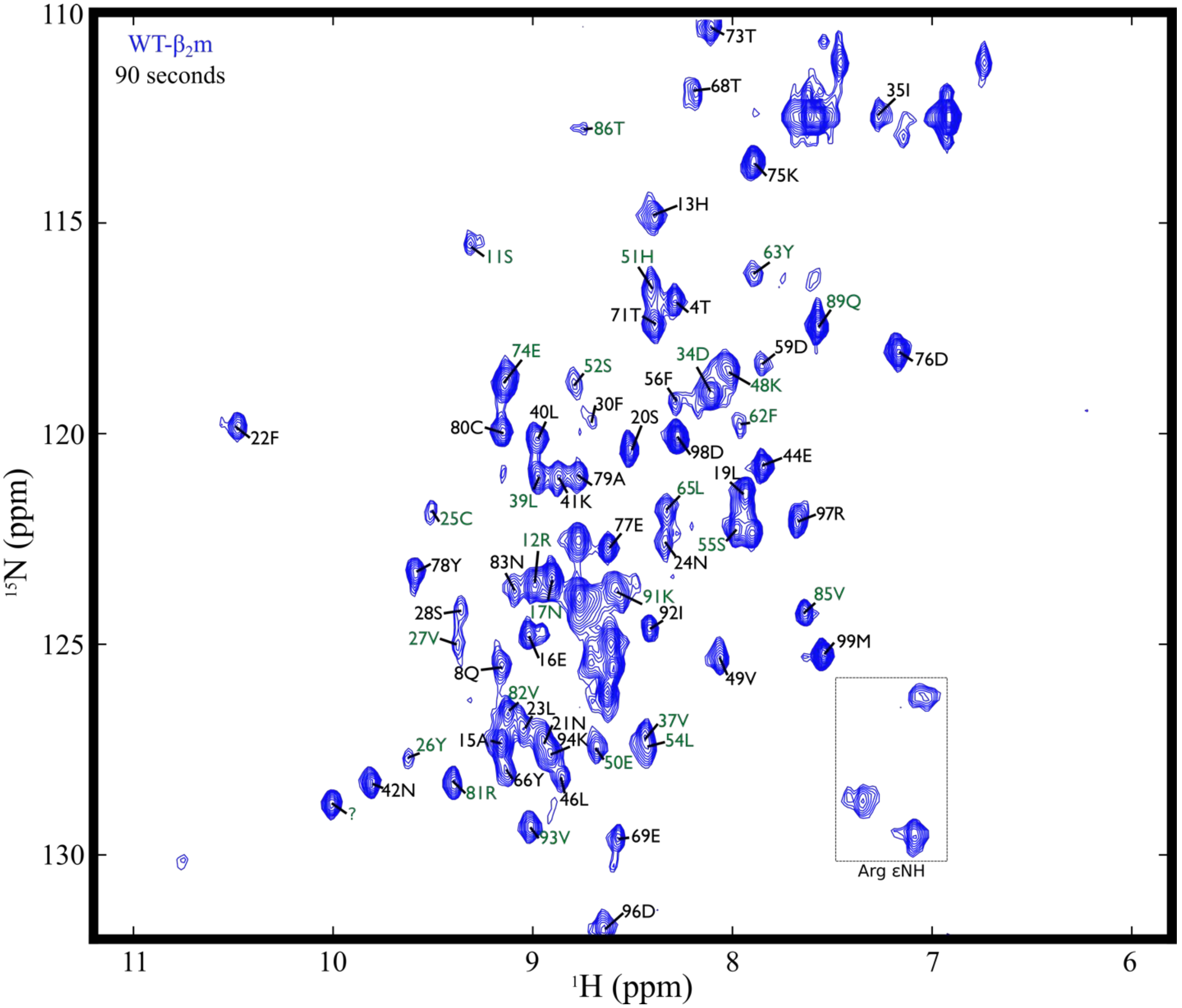

Supplementary Fig. S2. Real-time refolding of WT-β_2m, monitored by NMR spectroscopy after 90 sec.

This ^{1}H-^{15}N-SOFAST-HMQC spectrum (blue) was recorded for 60 sec, starting acquisition 30 sec after the initiation of the real-time refolding time course, in 167 mM sodium phosphate buffer pH 7.4, 20 °C with a final protein concentration of 300 μM. The I_T-state is the major species observed in the spectrum at this time (25) (while the N-state is populated to only ~14% as judged by spectral resonance intensities). The assignments shown were transferred from those of ΔN6- (BMRB: 17166) and WT-β_2m (BMRB: 17165), obtained under similar conditions (16). Some residues are not assigned due to spectral crowding or ambiguity in mapping from the reference ΔN6- and WT-β_2m assignments. A total of 69 residues were successfully assigned and 26 of them (green labels) were found to be unique to the I_T-state (i.e they do not overlap with resonances in the N-state) (Supplementary Fig. 6e). The only assigned peak missing in this spectrum is Gly43 which is folded in the ^{15}N dimension and therefore has negative intensity (only positive contours are shown in this figure).

Supplementary Figure S3

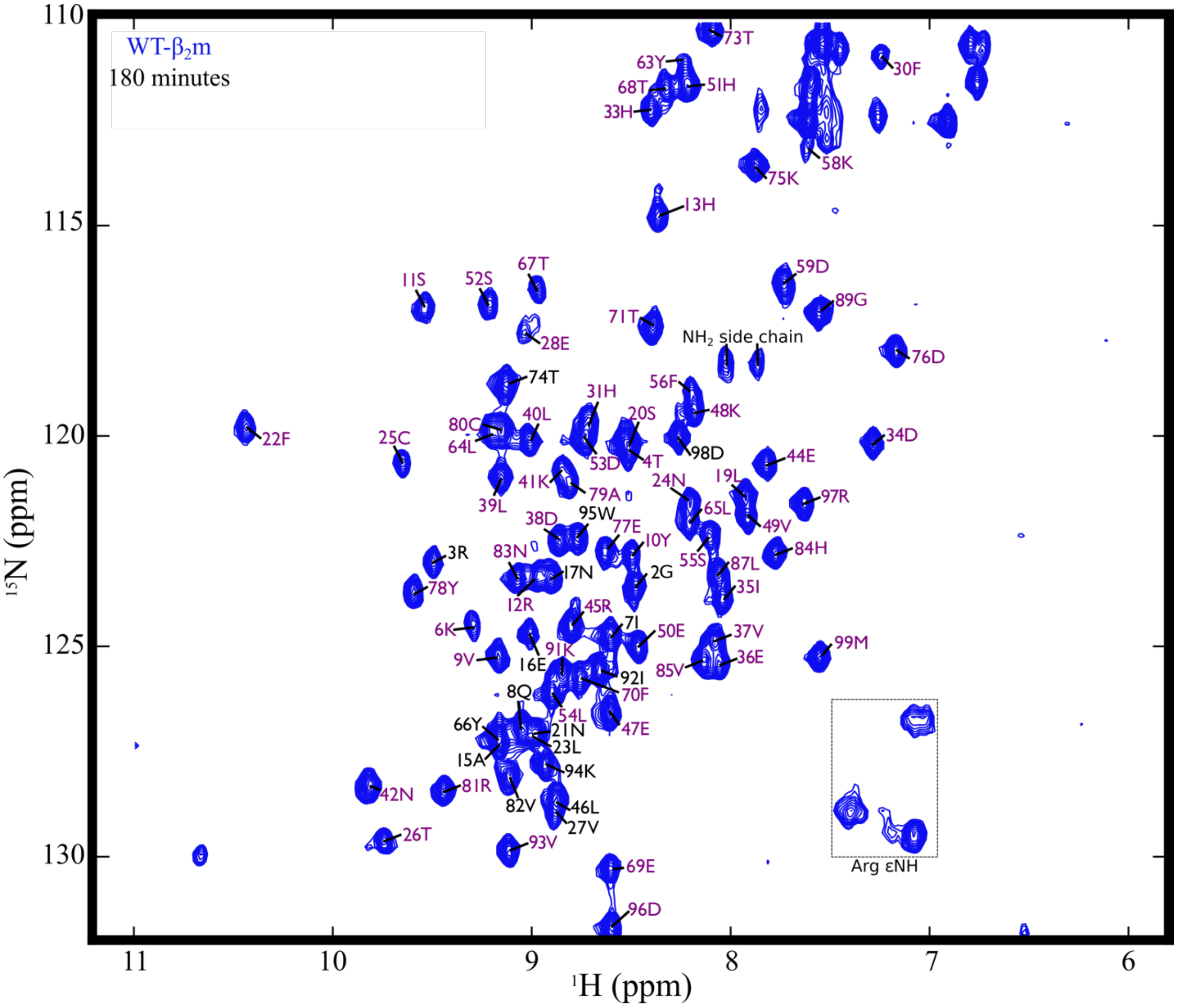

Supplementary Fig. S3. Real-time refolding of WT-β_2m, monitored by NMR spectroscopy after 180 min.

This ^{1}H-^{15}N-SOFAST-HMQC spectrum (blue) was recorded for 180 min in 167 mM sodium phosphate buffer pH 7.4, 20 °C with a final protein concentration of 300 μM. The WT-β_2m N-state is the only species observed in the spectrum. The assignments shown were transferred from those of WT-β_2m (BMRB: 17165), obtained under similar conditions (16). Some residues are not assigned due to spectral crowding and ambiguity in mapping from the reference WT-β_2m assignments. A total of 87 residues were successfully assigned and 70 of them (purple labels) were found to be unique to the N-state (i.e they do not overlap with resonances in the I_T state) (Fig. 4e). The only two assigned peaks missing in this spectrum are Gly18 and Gly43 which are folded in the ^{15}N dimension and therefore have negative intensity (only positive contours are shown in this figure).

Supplementary Figure S4

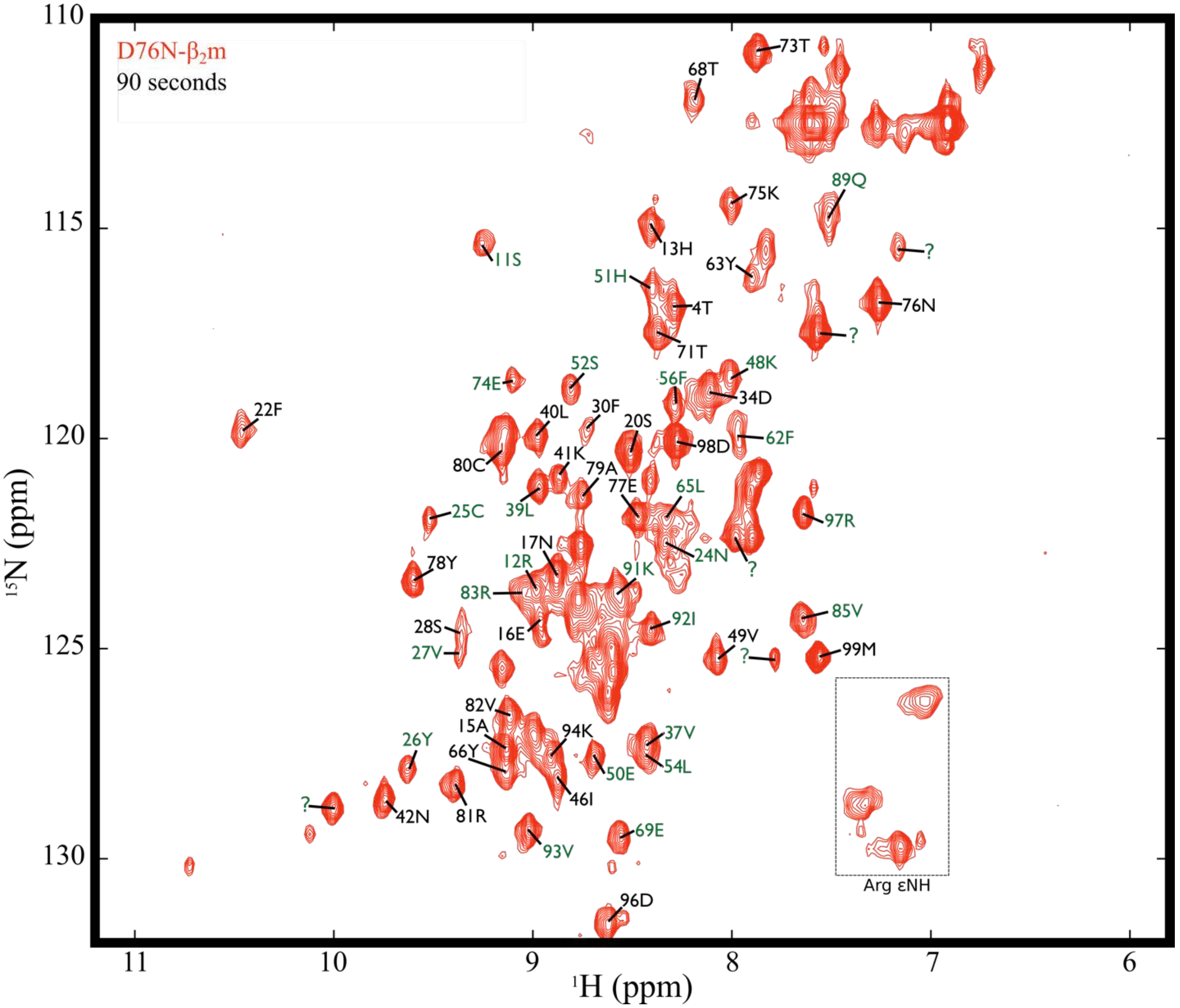

Supplementary Fig. S4. Real-time refolding of D76N-β_2m, monitored by NMR spectroscopy after 90 sec.

This ^{1}H-^{15}N-SOFAST-HMQC spectrum (red) was recorded for 60 sec, starting acquisition 30 sec after the initiation of the real-time refolding time course, in 167 mM sodium phosphate buffer pH 7.4, 20 °C with a final protein concentration of 300 μM. The I_T-state is the major species populated observed in the spectrum at this refolding time (25) (while the N-state is populated to only ~13% as judged by spectral resonance intensities). The assignments shown were transferred from those of ΔN6- (BMRB: 17166) (16) and D76N-β_2m (BMRB: 50302; see Experimental Procedures). Some residues are not assigned due to spectral crowding and ambiguity in mapping from the reference ΔN6- and D76N-β_2m assignments. A total of 58 residues were successfully assigned and 25 of them (green labels) were found to be unique to the I_T-state (i.e. they do not overlap with those of the N-state) (three of which could not be assigned and are indicated by "?") (Supplementary Fig. 6f). The only assigned peak missing in this spectrum is Gly43 which is folded in the ^{15}N dimension and has therefore negative intensity (only positive contours are shown in this figure).

Supplementary Figure 5

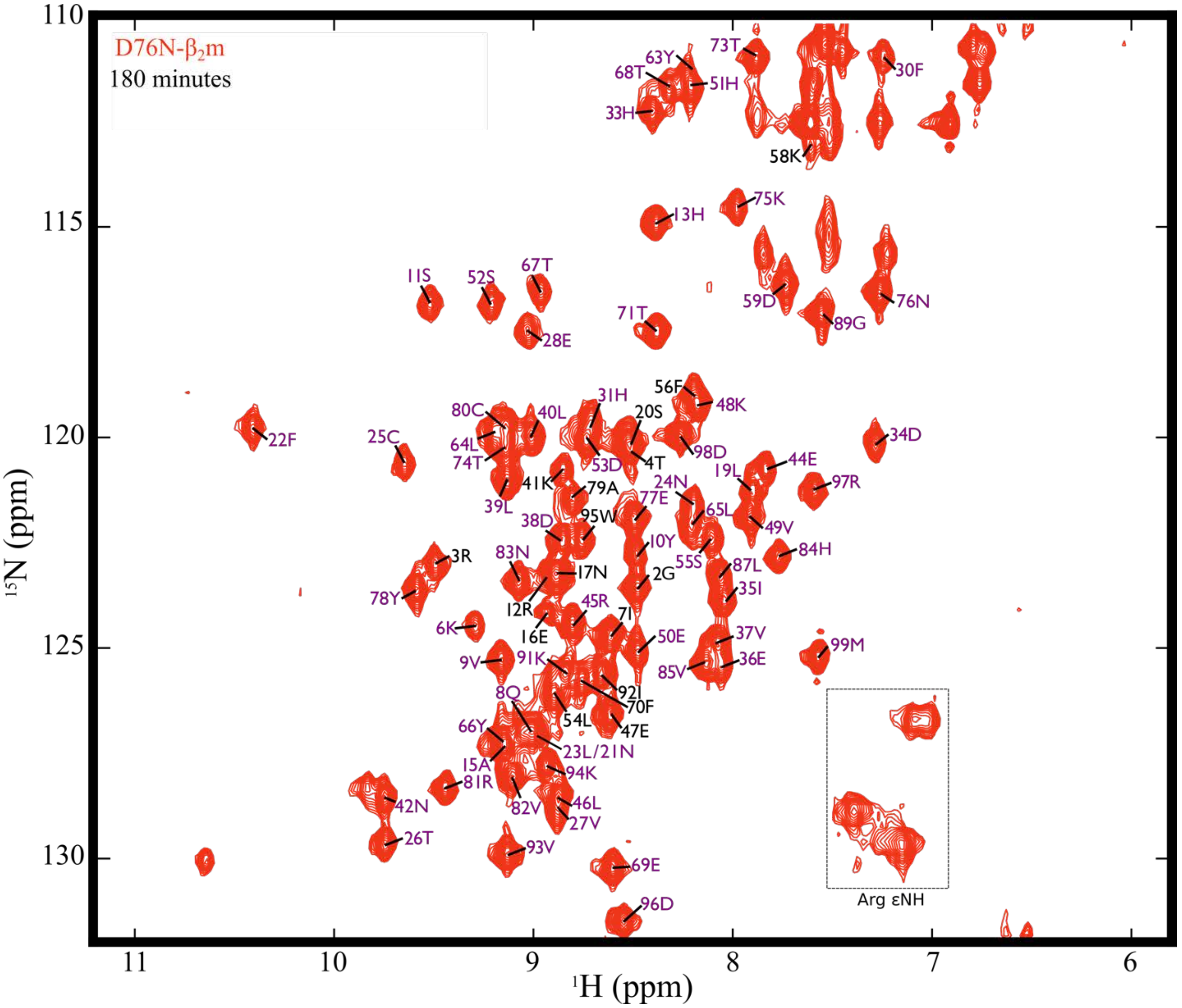

Supplementary Fig. S5. Real-time refolding of D76N-β_2m, monitored by NMR spectroscopy after 180 min.

This ^{1}H-^{15}N-SOFAST-HMQC spectrum (red) was recorded for 180 min in 167 mM sodium phosphate buffer pH 7.4, 20 °C with a final protein concentration of 300 μM. The D76N-β_2m N-state is the only species observed in the spectrum. The assignments shown were transferred from those of native D76N-β_2m, obtained under identical conditions (BMRB: 50302; see Experimental Procedures). Some residues are not assigned due to spectral crowding and ambiguity in mapping from the reference D76N-β_2m assignments. A total of 87 residues were successfully assigned and 66 of them (purple labels) were found to be unique to the N-state (i.e they do not overlap with resonances in the I_T state) (Fig. 4f). The only two assigned peaks missing in this spectrum are residues Gly18 and Gly43 which are folded in the ^{15}N dimension and therefore have negative intensity (only positive contours are shown in this figure).

Supplementary Figure S6

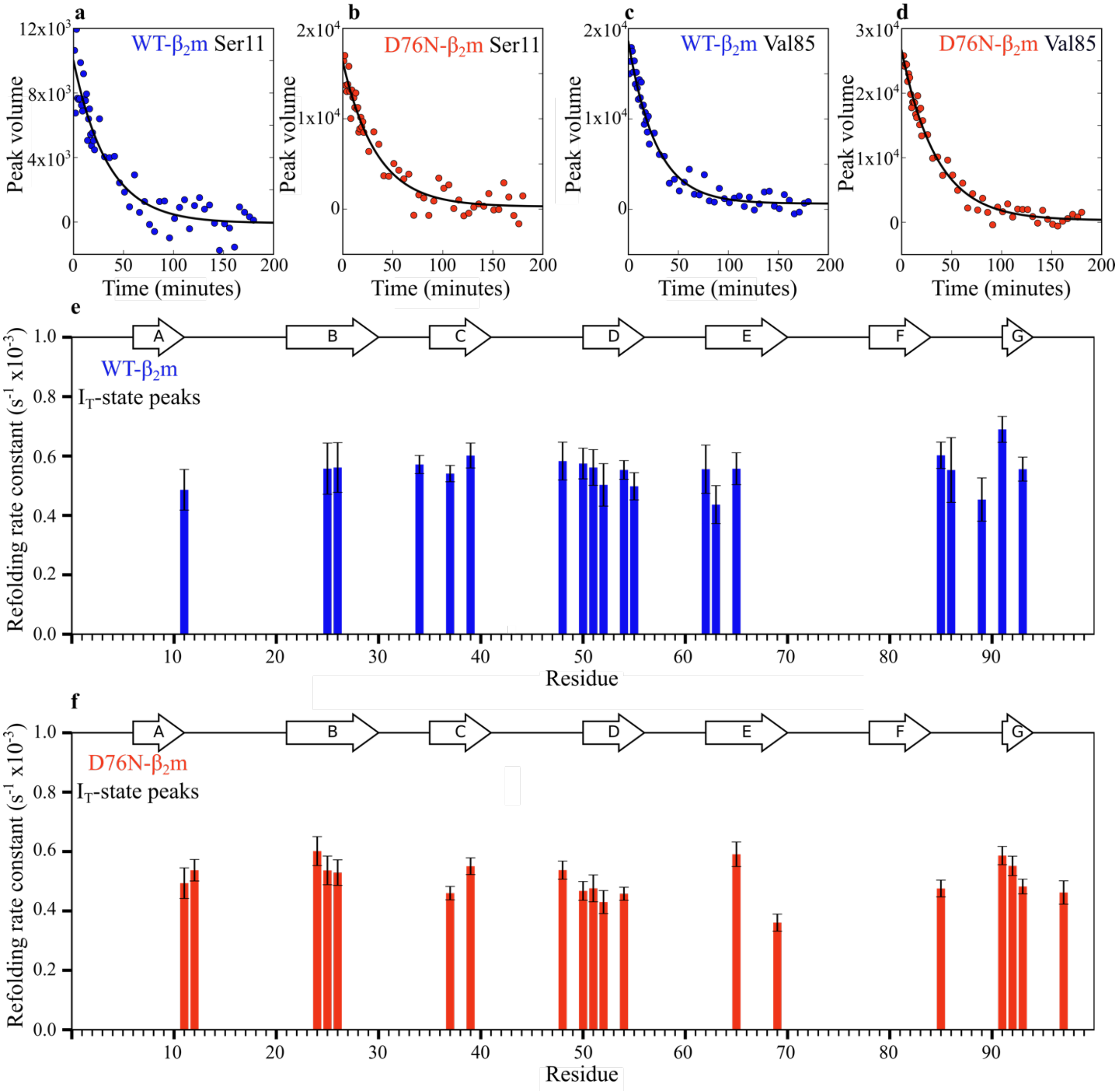

Supplementary Fig. S6. Single-residue refolding rates for I_T-state peaks of WT- and D76N-β_2m, monitored by NMR spectroscopy.

WT-β_2m is in blue and D76N-β_2m is in red. **(a-d)** Representative data and fits in black for the single residue refolding rates fitted to a single exponential. **(e, f)** The rate constants for individual residues that could be measured with confidence (where the error on the fit is no more than 3 median absolute deviations of all errors within each dataset) are shown in (e) and (f) for WT- and D76N-β_2m, respectively. Error bars are the fitting errors.
